# A Case of Ovarian Fibromatosis and Massive Ovarian Oedema Associated With Intra-Abdominal Fibromatosis, Sclerosing Peritonitis and Meig's Syndrome

**DOI:** 10.1080/13577140400011136

**Published:** 2004-12

**Authors:** Emma L. Spurrell, Yen C. Yeo, Terence P. Rollason, Ian R. Judson

**Affiliations:** 1 Sarcoma Unit Royal Marsden Hospital Fulham Road London UK; 2 Birmingham Women's Hospital Department of Histopathology Edgbaston Birmingham B15 2TG UK; 3 Department of Medical Oncology King George V Building, 1st Floor St Bartholomew's Hospital West Smithfield London EC1A 7BE UK

## Abstract

*Purpose:*To discuss a case of ovarian fibromatosis/massive ovarian oedema, intra-abdominal fibromatosis, sclerosing peritonitis and Meig's syndrome. To review the reported therapeutic options.

*Patients:* Case report of a 27-year-old female with the combined pathology of ovarian fibromatosis/massive ovarian oedema, intra-abdominal fibromatosis, sclerosing peritonitis and Meig's syndrome.

*Methods:* This patient was treated with supportive care and cytotoxic chemotherapy.

*Results:* Despite the benign nature of the ovarian pathology, this patient presented with life-threatening complications. Response to treatment was probably multi-factorial combining the effects of cytotoxics, use of steroids and good supportive care. She remains in complete remission 4 years post completion of chemotherapy.

*Conclusion:* There are reports in the literature of ovarian fibromatosis/massive ovarian oedema, luteinised thecomas, intraabdominal fibromatosis and Meig's syndrome occurring together in a variety of combinations. Treatment has been described with radiotherapy, cytotoxic and non-cytotoxic chemotherapy regimens. This case provides a link between ovarian fibromatosis/massive ovarian oedema, intra-abdominal fibromatosis, sclerosing peritonitis and Meig's syndrome not previously described.


*Sarcoma*, December 2004, *VOL. 8, NO. 4, 113–121*

Taylor & Francis healthsciences

ORGINAL ARTICLE

### A case of ovarian fibromatosis and massive ovarian oedema associated with intra-abdominal fibromatosis, sclerosing peritonitis and Meig's syndrome

EMMA L. SPURRELL[1], YEN C. YEO[2], TERENCE P. ROLLASON[2] & IAN R. JUDSON[1]

[1]*Sarcoma Unit, Royal Marsden Hospital, Fulham Road, London, UK;* [2]*Birmingham Women's Hospital, Department of Histopathology, Edgbaston, Birmingham B15 2TG, UK*

**Abstract**

*Purpose:* To discuss a case of ovarian fibromatosis/massive ovarian oedema, intra-abdominal fibromatosis, sclerosing peritonitis and Meig's syndrome. To review the reported therapeutic options.

*Patients:* Case report of a 27-year-old female with the combined pathology of ovarian fibromatosis/massive ovarian oedema, intra-abdominal fibromatosis, sclerosing peritonitis and Meig's syndrome.

*Methods:* This patient was treated with supportive care and cytotoxic chemotherapy.

*Results:* Despite the benign nature of the ovarian pathology, this patient presented with life-threatening complications. Response to treatment was probably multi-factorial combining the effects of cytotoxics, use of steroids and good supportive care. She remains in complete remission 4 years post completion of chemotherapy.

*Conclusion:* There are reports in the literature of ovarian fibromatosis/massive ovarian oedema, luteinised thecomas, intra-abdominal fibromatosis and Meig's syndrome occurring together in a variety of combinations. Treatment has been described with radiotherapy, cytotoxic and non-cytotoxic chemotherapy regimens. This case provides a link between ovarian fibromatosis/massive ovarian oedema, intra-abdominal fibromatosis, sclerosing peritonitis and Meig's syndrome not previously described.

### Presentation of case

AP is a 27-year-old female who presented to her local accident and emergency department in July 1999 with a history of bloating, abdominal pain and altered bowel habit. On examination she was found to have an abdominal mass and clinical ascites. An ultra-sound scan confirmed bilateral ovarian enlargement with significant ascites. Beta-HCG, CA-125 and alpha-fetoprotein were within normal range. Past medical history was significant for a termination of pregnancy 2 years previously and a renal calculus. There was no history of colonic polyps and no significant family history. The patient was taking the combined oral contraceptive at the time of presentation.

A laparotomy with bilateral ovarian biopsies was undertaken. At laparotomy, 4.5 l of ascites were drained and the abdominal structures appeared normal.

The histology from the ovarian biopsies was thought to be consistent with a sclerosing stromal tumour of the ovary. Bilateral salpingo-oophorectomy and infra-colic omentectomy was then undertaken.

Following the second laparotomy the patient became increasingly symptomatic with worsening ascites and vomiting. A barium follow-through revealed incomplete small bowel obstruction in the pelvis as well as a 15 cm stricture in the sigmoid colon thought to be due to extrinsic compression. A flexible sigmoidoscopy was performed and biopsies taken which revealed inflammation only. The subacute bowel obstruction was initially managed conservatively and the patient commenced total parenteral nutrition (TPN).

At the end of July 1999 a third laparotomy was undertaken for persistent small bowel obstruction and clinical evidence of recurring mass in the abdomen. At laparotomy the entire pelvis and lower abdomen were involved in a retroperitoneal mass matting the bowel together. Biopsies were taken from the retroperitoneal mass, the omentum and the sigmoid colon.

Correspondence to: Dr E Spurrell, Department of Medical Oncology, King George V Building, 1st Floor, St Bartholomew's Hospital, West Smithfield, London EC1A 7BE, UK. Fax: +44-20-7786-9414; E-mail: emma.spurrell@icr.ac.uk

1357-714X print/1369–1643 online © 2004 Taylor & Francis Ltd
DOI: 10.1080/13577140400011136

The original ovarian histology was reviewed along with the new biopsies. Both ovaries were reported as showing central stromal oedema with a zone of increased cortical cellularity but sparing of the native structures, there were few mitoses and both ovaries showed evidence of torsion. An incidental cystadenoma was noted in the right ovary. The overall appearance was considered consistent with ‘immature fibromatosis’ and massive ovarian oedema. The retroperitoneal histology showed fibroblastic proliferations composed of long fascicles of spindle cells invading the adipose tissue. Reactive mesothelial cells and fibrinous exudates were seen on the surface. The appearance was predominantly that of intra-abdominal fibromatosis for which the superficial areas showed indistinguishable sclerosing peritonitis. The omental and sigmoid biopsies were consistent with non-specific inflammation.

The patient was then transferred to the Royal Marsden Hospital (RMH) for consideration of chemotherapy for inoperable disease. On admission to RMH the patient was clinically stable but requiring on-going TPN for persistent small bowel obstruction. Baseline CT scan showed an extensive plaque of peritoneal tumour, loculated ascites and gross compression of the stomach, small and large bowel. There were large bilateral pleural effusions.

In August 1999 she received a first cycle of chemotherapy with doxorubicin and ifosfamide. Four days following treatment she became acutely hypoxic, tachycardic and oliguric. On transfer to the High Dependency Unit (HDU) she was febrile, pulse 140 bpm, BP 130/80, $O_2$ saturation 78% on room air. She was treated for aspiration pneumonia and anti-coagulated for possible pulmonary embolus. A large left pleural effusion was drained. She responded well to antibiotics and fluid resuscitation and was returned to the ward 3 days later. An episode of neutropenic sepsis occurred during the following 2 days but was managed without complication.

A second cycle of chemotherapy was given on schedule. Ifosfamide was replaced by DTIC due to ifosfamide encephalopathy with the first cycle. Two cycles of doxorubicin/DTIC were given and re-imaging showed a partial remission of the peritoneal disease. However, contrast imaging of the GI tract revealed persistent high small bowel obstruction. TPN was continued. An attempted small bowel stent procedure failed.

Liver function became increasingly deranged. Doxorubicin/DTIC were, therefore, replaced by vinblastine and methotrexate for cycle 4. Cycles 5 and 6 comprised vinblastine with cyclophosphamide, treatment being completed in November 1999. Post treatment re-staging revealed a significant reduction in both the peritoneal disease and the pleural effusions. Unfortunately the patient was re-admitted to HDU for an episode of sepsis requiring inotropic support but recovered well.

Following completion of chemotherapy, the obstructive GI symptoms persisted and the patient remained dependent upon TPN. By March 2000 this appeared to be resolving slowly and NG feeding was instituted. Her bowels started opening and TPN was eventually stopped. Liver function remained persistently deranged. Hepatitis screen was negative. A liver biopsy was eventually undertaken which showed exogenous iron overload and cholestasis consistent with TPN. HFE genotyping was not consistent with haemochromatosis.

In May 2000, 11 months following admission to the referring hospital, the patient was discharged home. Imaging at this time showed virtual complete resolution of effusions, small volume ascites, significant reduction in peritoneal disease, hepatosplenomegaly and no evidence of GI obstruction.

Following discharge a large pericardial effusion required drainage and a window procedure in August 2000. A rectovaginal fistula has been noted which is relatively asymptomatic and under surveillance only. An abdominal scar revision was successfully undertaken by the plastic surgeons in June 2001 for cosmetic reasons. Otherwise, liver function has normalised, the patient has gained weight (though remains below ideal body weight) and has returned to normal active life. The most recent CT scan in February 2003 showed continued complete remission 4 years following completion of chemotherapy.

Figures 1–4 show the biopsy specimens taken from the case presented. They demonstrate the histopathological changes seen in massive ovarian oedema, ovarian fibromatosis, sclerosing peritonitis and intra-abdominal fibromatosis. Figure 5 is taken from pre-chemotherapy CT images which show the dense mass of solid intra-abdominal fibromatosis. Figure 6 is the most recent CT image showing complete resolution of the intra-abdominal fibromatosis and normal intra-abdominal structures.

### Introduction

Fibromatosis describes a group of fibrous tissue proliferations that, although benign, can be locally invasive but do not metastasise.[1] X-chromosome inactivation molecular analyses have confirmed these tumours to be clonal, thus identifying them as neoplasms and not products of an inflammatory response.[2] Similarly, molecular analysis has shown recurrent fibromatosis within an individual to be derived from the same cell clone as the primary lesion.[2] The fibromatoses have a predilection for certain anatomic sites and are divided into two types – superficial and deep.[1] Superficial fibromatoses arise from fascia or aponeuroses, rarely involve deep structures and are small and slow growing.[1]

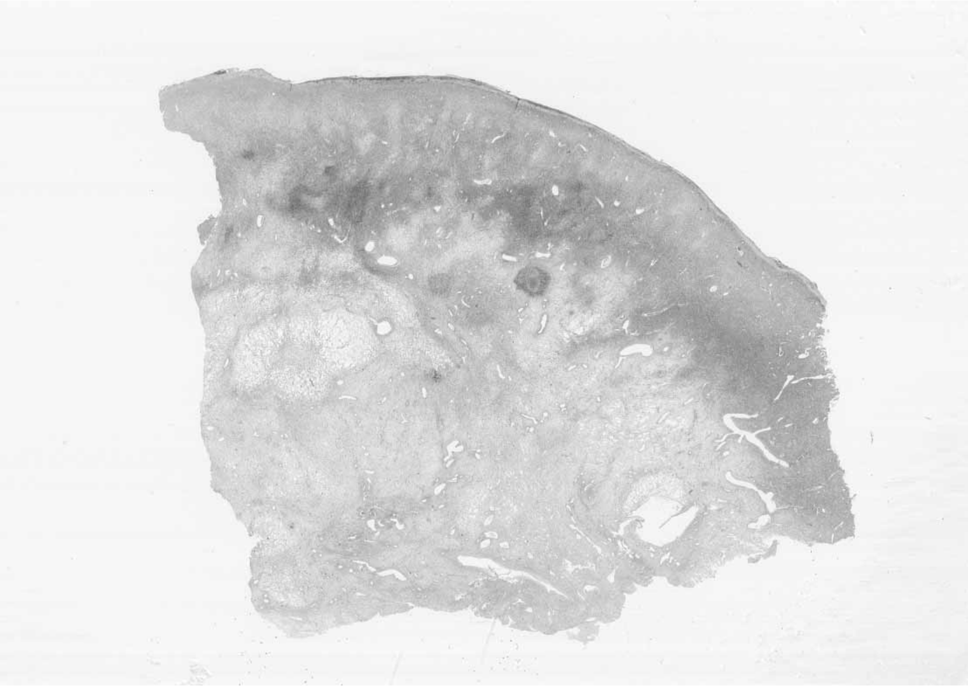

**Fig. 1.** *Ovary showing a zone of increased cortical cellularity with central stromal oedema, typical of massive ovarian oedema.*

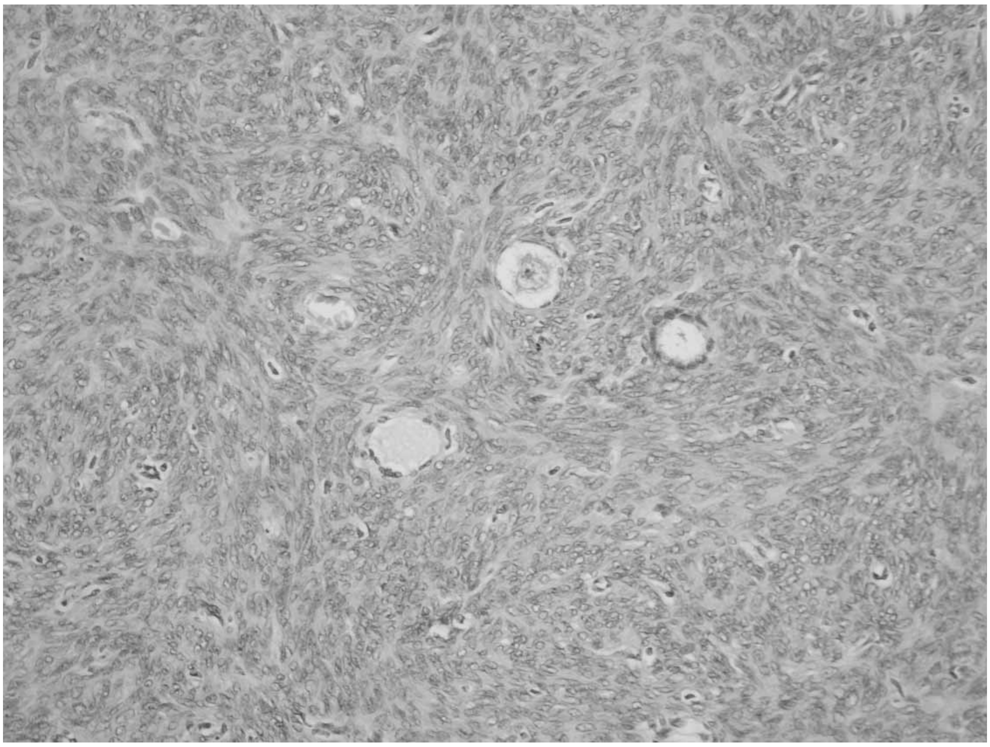

**Fig. 2.** *The ovarian cellular cortex is composed of a spindle cell proliferation with entrapment of numerous ovarian structures consistent with ovarian fibromatosis.*

Deep fibromatoses principally involve the deep muscles of the trunk and extremities (including the abdomen and pelvis) and tend to be more aggressive with a high recurrence rate.[1] Deep fibromatoses are also referred to as desmoid tumours. Intra-abdominal fibromatosis is divided into pelvic, mesenteric, retroperitoneal and intra-abdominal desmoid tumours associated with Gardner's Syndrome.[1] Gardner described the association of familial intestinal polyposis, osteomas, fibromas and epidermal or sebaceous cysts in 1951.[1] This is an autosomal dominant condition not associated with the case under discussion.

This is a case of immature ovarian fibromatosis associated with massive ovarian oedema, intra-abdominal involvement, sclerosing peritonitis

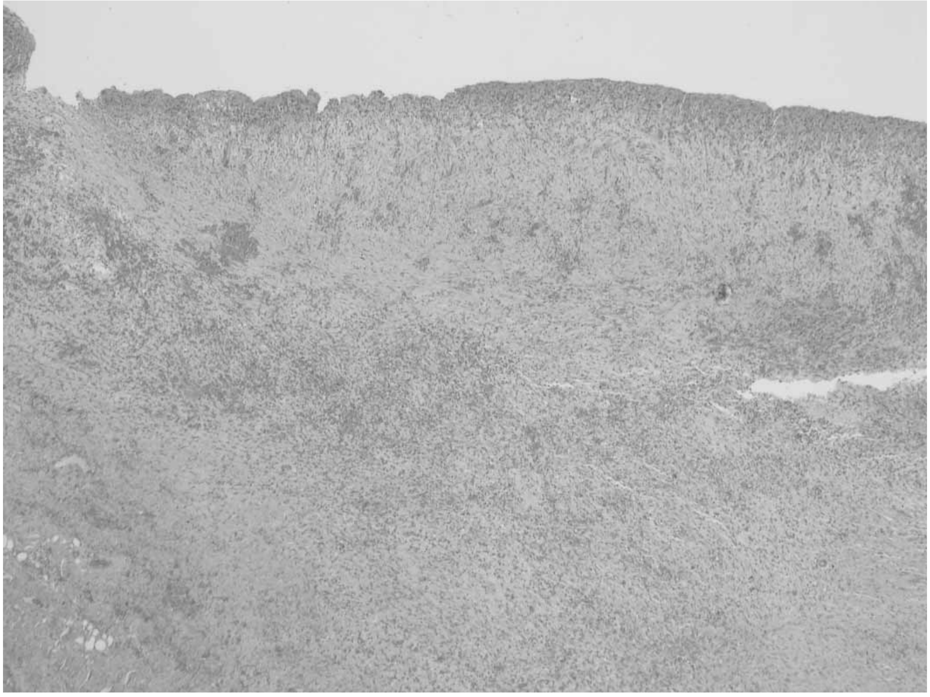

**Fig. 3.** *Proliferation of mesothelial cells associated with fibrinous exudates and fibrosis are seen on the surface of the retroperitoneal biopsy, features of sclerosing peritonitis.*

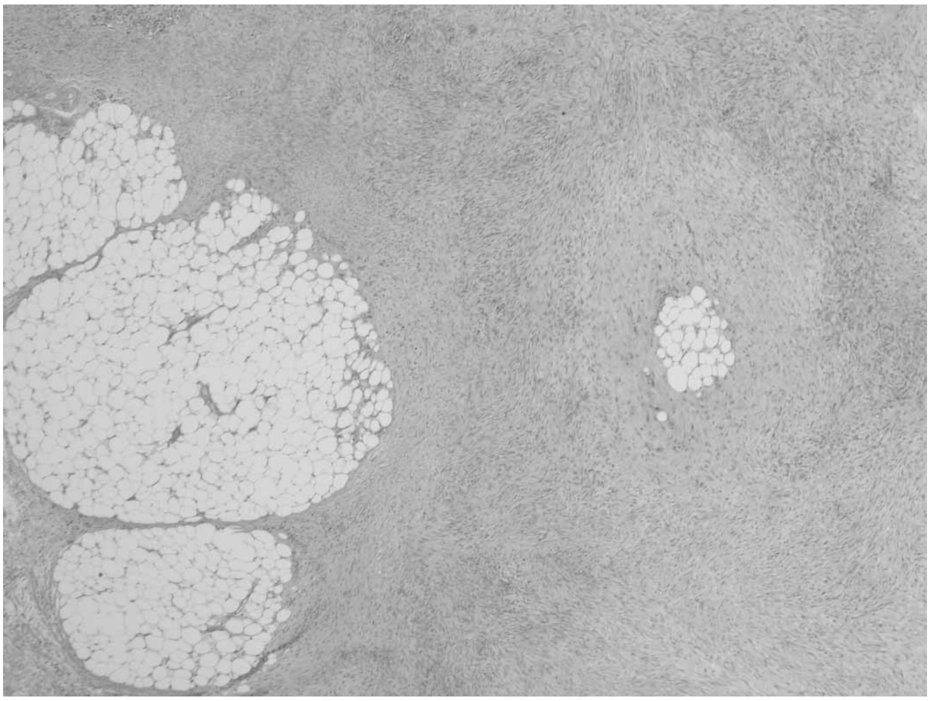

**Fig. 4.** *Long fascicles of spindle cells infiltrating underlying adipose tissue from the retroperitoneal biopsy consistent with intra-abdominal fibromatosis.*

and probable Meig's syndrome. Immature highly cellular fibroblastic tumour-like lesions of the ovary have been described in the literature as 'thecomas', 'fibromatosis', 'fibrothecomas' and 'luteinised thecomas'.[3] Thecomas and fibromas represent tumours of the ovarian stroma. Thecomas are histologically composed of lipid-containing cells and fibromas almost entirely of spindle cells. There is, however, considerable histological overlap between the two, such that intermediate tumours are often termed fibrothecomas.[4] Luteinised thecomas are composed of nests of pale luteinised cells within a background population of spindle cells and are known to be associated with reactive peritoneal proliferations showing fibrosis, surface fibrin, chronic inflammation and focal mesothelial hyperplasia known

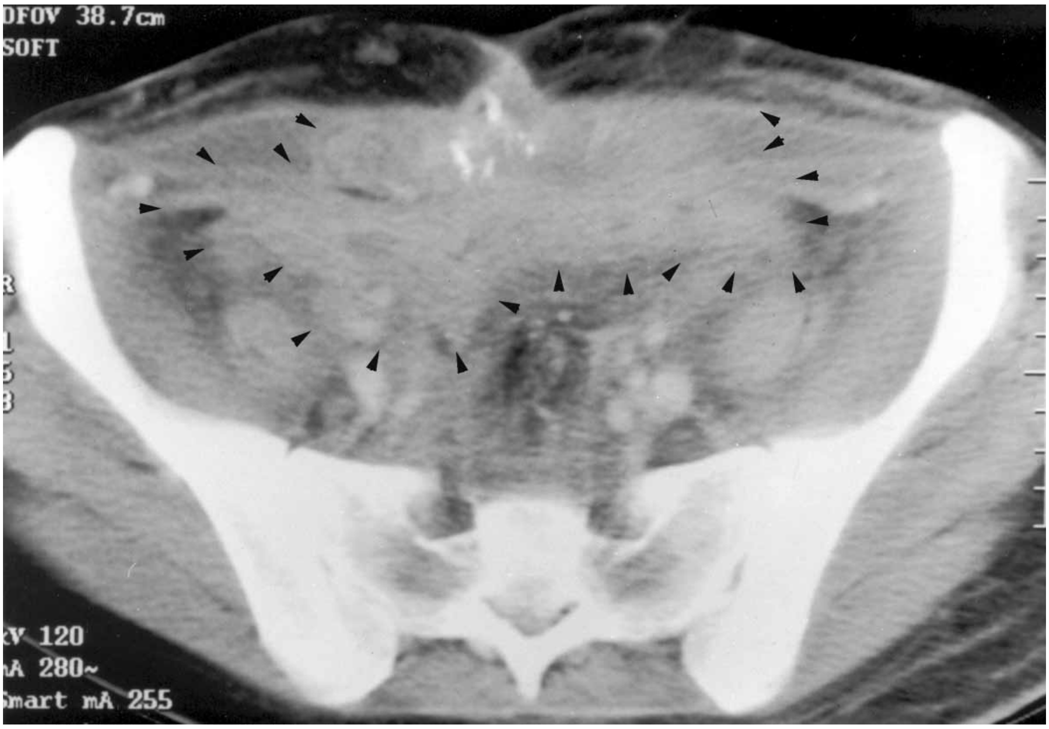

**Fig. 5.** *Baseline CT taken in August 1999 showing dense fibrosis within the abdomen. The main tumour plaque has been outlined by arrowheads. There are no visible gas-filled bowel loops due to persistent small bowel obstruction and physical compression of the bowel by tumour.*

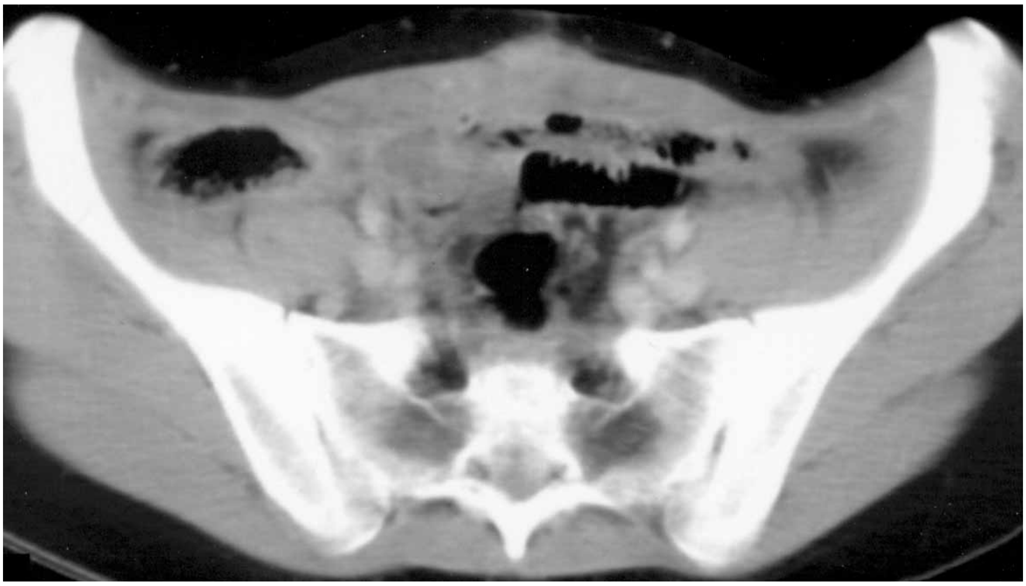

**Fig. 6.** *CT taken in February 2003 showing resolution of the dense abdominal fibromatosis. There are visible normal gas-filled loops of bowel as the GI tract is no longer obstructed.*

as sclerosing peritonitis. Luteinised thecomas closely resemble immature fibromatosis but are usually unilateral and oestrogenic, unlike fibromatosis that is endocrinologically inactive and often bilateral.[3] Common to all these conditions is their young age at onset, presentation with abdominal/pelvic pain, pelvic swelling, occasional association with ascites and pleural effusions and with massive ovarian oedema.[3] There are also reported associations with exposure to antiepileptic drugs[3,5] and beta-blockers.[3,6]

Massive ovarian oedema describes the accumulation of fluid within the ovarian stroma.[7] It is said to occur in younger females (average age 19–22 years), causes abdominal/pelvic pain and may be associated with hormonal irregularities causing menstrual disturbance, virilisation and occasionally precocious puberty.[7,8] It is usually a unilateral phenomenon, the right ovary being affected more often than the left.[8] One theory pertaining to the pathogenesis of massive ovarian oedema suggests that there is disruption of ovarian lymphatic and venous drainage due to intermittent partial torsion.[7,8] An analysis of 18 cases of massive ovarian oedema showed that in 14 there was torsion of the ovaries.[8] In favour of this theory is the observation that the ovaries revert to normal size once the torsion has been corrected by ovarian suspension.[8] The relationship between ovarian fibromatosis and massive ovarian oedema is not clear. A study of 25 patients with ovarian enlargement in 1984 showed that 14 cases had fibromatosis as the predominant pathology, six of which also had massive ovarian oedema; 11 cases showed massive ovarian oedema as the predominant pathology, eight of which contained foci of fibromatosis; seven of the 25 cases showed evidence of ovarian torsion.[9] It would appear that massive ovarian oedema is associated with both fibromatosis and ovarian torsion, but which comes first the torsion or the fibromatosis?

There are two theories to explain the relationship between ovarian fibromatosis and torsion. The first states that the primary pathology is fibromatosis that causes ovarian torsion and then massive ovarian oedema.[3] The second theory states that torsion itself causes fibromatosis by stimulating local platelets and macrophages to secrete growth factors that induce massive fibroblastic proliferation and oedema.[3] It is thought that patients presenting in the acute phase of this process exhibit a mitotically active ‘immature’ fibromatosis (as in the case discussed here) and those presenting in the resolving phase exhibit a ‘mature’ sclerotic fibromatosis.[3]

Meig’s Syndrome describes the combination of ovarian fibroma associated with ascites and hydrothorax.[10] Meig’s syndrome is mentioned due to the otherwise unexplained massive pleural effusions seen in our patient and other similar cases in the literature. It is thought that the ovarian tumour produces ascitic fluid that migrates to the pleural cavity via diaphragmatic lymphatics. The effusions and ascites are usually transudative but blood stained ascites is seen in cases associated with ovarian torsion. Characteristic of the syndrome is the disappearance of the ascites and hydrothorax following resection of the ovarian tumour.

The case presented represents the previously undescribed occurrence of sclerosing peritonitis in association with ovarian fibromatosis/massive ovarian oedema (previously, sclerosing peritonitis has only been reported in association with luteinised thecomas). The pleural and pericardial effusions in association with ascites suggests associated Meig’s syndrome. Our patient also showed radiological and biopsy proven evidence of intra-abdominal fibromatosis, or desmoid, a poorly understood phenomenon very rarely reported to occur in association with ovarian fibromatosis. The management of the fibromatoses is dependent upon the site of disease and its associated morbidity, but data are scanty and varied, as discussed below.

### Management

Discussed below is a review of the literature concerning the management of fibromatosis. Information relating specifically to the above case is anecdotal, therefore, included is discussion regarding management of fibromatosis in general. When referring to intra-abdominal disease, this includes pelvic disease. The literature discussed below is limited, where possible, to articles concerned mainly with intra-abdominal disease (as opposed to trunk and extremity) and not related to Gardner’s syndrome.

### *Surgery*

In the first instance, surgical resection of abdominal desmoid tumours is the treatment of choice where possible. Complete excision of abdominal desmoid tumours is often difficult due to their close proximity to vital structures. There is debate in the literature as to whether positive or negative surgical margins influence local recurrence rates. A review of 130 patients with retroperitoneal soft tissue sarcomas showed a local recurrence rate of 63% post local excision versus 39% following a wide resection.[11] Similarly, a retrospective review of 189 patients with desmoid tumours, seven of which were intra-abdominal, showed a 10 year post-operative relapse rate of 27% in patients with negative surgical margins and 54% in those with positive margins.[12] A meta-analysis of 22 published analyses included 845 patients with desmoid tumours, 60 of which were intra-abdominal, and showed a local control rate of 72% with negative surgical margins versus 41% with postive margins.[13] Alternatively, an analysis of 103 patients with trunk and extremity desmoid tumours, none of which were intra-abdominal, showed a local recurrence rate of 22% in those with positive resection margins and 24% in those with negative margins.[14] They suggest that surgery that preserves structure and function should be the goal, not negative resection margins.[14]

### *Radiotherapy*

The literature pertaining to radiotherapy for desmoid tumours mainly involves extremity or abdominal wall tumours. Information regarding radiotherapy to intra-abdominal tumours is minimal. The meta-analysis of 22 articles mentioned above compared surgery versus radiotherapy for patients with desmoid tumours (excluded in this analysis were patients with Gardner’s).[13] Cases of intra-abdominal fibromatosis represented 7% of the total number of patients analysed in this publication. This paper suggested that the addition of radiotherapy to surgery improved local control rates for post-operative negative surgical margins (72 vs. 94% with addition of radiotherapy) and positive surgical margins (41 vs. 75% with addition of radiotherapy).[13] Similar findings were published in the retrospective review of 189 cases of desmoid tumours also mentioned above, whereby optimum management was considered to be surgery with negative margins but adjuvant radiotherapy considerably offset the effect of positive surgical margins (10-year relapse rate with positive margins, 54 vs. 25% with addition of radiotherapy).[12]

In these large analyses, intra-abdominal desmoid tumours are significantly under-represented making it difficult accurately to extrapolate the results to these tumours. Intra-abdominal disease is often difficult to irradiate due to the large field required, and radiotherapy carries the potential for gastro-intestinal side effects in patients potentially already experiencing GI morbidity. A published analysis of 54 desmoid tumours treated with surgery and radiotherapy reported a sub-analysis of the six abdominal tumours within the cohort, and found that there was an increased frequency of treatment failure in the abdominal tumours.[15] Their explanation for this was that the abdominal tumours were more likely to have spread further making it difficult to encompass all of the disease within the radiotherapy field.[15]

*Non-cytotoxic chemotherapy*

Hormonal treatment for desmoid tumours is the major non-cytotoxic chemotherapy reported in the literature. Abdominal wall desmoid tumours have been noted to occur more commonly in young women, pregnant women or in the first year following gestation.[1] For this reason, it is believed possible that tumour growth is under the influence of oestrogen and progesterone. Oestrogen receptors and anti-oestrogen binding sites have been demonstrated in desmoid tumours.[16] A study of 15 desmoid tumours found oestrogen receptors to be present in 33%, with equal incidence in males and females but higher receptor content in females.[16] Anti-oestrogen binding sites were found in 79%.[16]

Other non-cytotoxic agents include non-steroidal anti-inflammatory drugs (NSAIDs) and glucocorticoids. Use of these agents originated from mouse tumour models, showing inhibition of tumour growth in the absence of products of arachidonic acid metabolism as a result of phospholipase A2 inhibition.[17] Glucocorticoids are known to inhibit phospholipase A2. NSAIDs reduce arachidonic acid metabolism via inhibition of cyclo-oxygenase.

Table 1 is a summary of the literature pertaining to intra-abdominal desmoid in the absence of Gardner's syndrome. Much of the data are anecdotal and many of the agents have been given in combination, making it difficult to isolate efficacy. The main hormonal agents used with some success are tamoxifen and progesterone.[18–22] Others include LHRH analogues, medroxyprogesterone acetate and toremifene (a tamoxifen derivative).[23,24] There are anecdotal reports of use of glucocorticoids and NSAIDs in combination with tamoxifen.[19–21]

There are two identifiable reports of non-cytotoxic agents used in cases very similar to the one presented in this report. The first involves a case of ovarian fibromatosis associated with diffuse intra-abdominal fibromatosis treated with tamoxifen and iv steroid resulting in complete resolution of bowel obstruction.[20] The second report includes a case of luteinised thecoma of the ovary with associated scerosing peritonitis.[25] The sclerosing peritonitis, which persisted following resection of the ovaries, was treated with i.v. hydrocortisone resulting in resolution of ascites and symptoms.[25] This patient was successfully re-challenged with hydrocortisone 2 months later when the disease flared.[25]

*Cytotoxic chemotherapy*

Despite the benign classification of fibromatosis, there has been success in treating unresectable tumours with chemotherapy. Table 2 is a summary of the different regimens that have been employed

**Table 1.** *Non-cytotoxic chemotherapy use in desmoid tumours (RR, response rate; CR, complete response; PR, partial response; SD, stable disease)*

| Year of publication | Number of patients | Gardner's syndrome | Number of abdominal tumours | Agent | Response |
|---|---|---|---|---|---|
| 1990[23] | 1 | No | 1 | LHRH (Zoladex) + medroxyprogesterone acetate | PR |
| 1991[18] | 1 | No | 1 | Tamoxifen | CR |
| 1996[19] | 1 | No | 1 | Tamoxifen + sulindac | PR |
| 1993[20] | 1 | No | 1 | Tamoxifen + IV steroid | PR |
| 1995[21] | 1 | No | 1 | Tamoxifen + prednisolone | PR |
| 1994[24] | 1 | No | Retroperitoneal | Toremifene | PR |
| 1983[22] | 11 | No | Unstated | Progesterone | RR 54.5%, SD 18% |

**Table 2.** *Cytotoxic chemotherapy regimens used over the years as published in the literature (CR, complete response; PR, partial response; MR, minor response; SD, stable disease; PD, progressive disease.)*

| Year of publication | Number of patients | Gardner's syndrome | Number of abdominal tumours | Regimen | Response |
|---|---|---|---|---|---|
| 1987[36] | 6 | Not stated | 2 | Vincristine/actinomycin-D/ cyclophosphamide | 4 CR, 1 PR |
| 1989[37] | 8 | No | 1 | Vinblastine/methotrexate (weekly) | 2 CR, 6 PR |
| 1993[38] | 12 | 4 | 4 | Doxorubicin/DTIC (5 cycles) | 2 CR, 4 PR, 2 SD (in the abdominal tumours: 2 CR, 2 PR) |
| 1999[28] | 17 | No | 0 | Vinorelbine/methotrexate (weekly) | 3 CR, 7 PR, 2 MR, 3 SD, 2 PD |
| 2002[39] | 7 | 4 | 6 | Cyclophosphamide/ doxorubicin OR ifosfamide/etoposide OR mitomycin/doxorubicin/cisplatin | 3 CR/PR |

over the years. Many of the regimens are doxorubicin-based with good results, but are limited by toxicity following cumulative dosing. Weekly methotrexate and vinblastine is an effective regimen with minimal toxicity, enabling patients to remain on treatment for prolonged periods of time (longest 12 months).[26,27] Peripheral neuropathy can be a treatment-limiting toxicity with this regimen that has been successfully overcome by substituting vinblastine for vinorelbine with similar good results.[28]

Limiting all of the studies are the small patient numbers and variation in regimens between studies and occasionally within studies. Importantly, as well as objective responses being reported, many articles have also mentioned the significant palliation of symptoms achieved with cytotoxic chemotherapy.

### Conclusion

The case presented here represents the rare combined occurrence of ovarian fibromatosis and massive ovarian oedema together with sclerosing peritonitis, intra-abdominal fibromatosis and Meig's syndrome. The origin of the extra-ovarian intra-abdominal pathology reported in this case, and other similar cases, is debated. In some reports, biopsies of extra-ovarian sites have revealed nodules of tumour similar to that seen in the resected ovaries postulating that cells may have become detached from the ovarian tumour and migrate into the peritoneal cavity by way of the ascites to implant elsewhere.[5,6,20,29] Other reports have described a generalised sclerosing peritonitis associated with ovarian luteinised thecomas and it is suggested that the ovarian tumour may be producing a fibrosis-inducing substance.[6,30–33] Against the latter argument is the observation that in many cases the peritoneal pathology progresses despite resection of the ovarian tumours, some patients requiring multiple laparotomies for recurrent small bowel obstruction.[6,25,30,34]

Despite the benign nature of the ovarian tumours, there is significant associated morbidity. The intra-abdominal disease frequently causes recurrent problems with small bowel obstruction sometimes requiring multiple resections. Fatal pulmonary emboli have been reported twice in the literature.[29,30] Both occurred post laparotomy for small bowel obstruction and may represent the increased thrombotic risk associated with the post-operative state or possibly a hypercoagulable state associated with the intra-abdominal pathology.

In conclusion, there are anecdotal reports in the literature of ovarian fibromatosis/massive ovarian oedema, intra-abdominal fibromatosis, luteinised thecomas, sclerosing peritonitis and Meig's syndrome occurring together in a variety of combinations.[4–6,9,20,25,29–34] Previously in the literature, sclerosing peritonitis has only been reported to occur in the presence of ovarian luteinised thecomas. Our case is unique being the first to describe the occurrence of sclerosing peritonitis in association with ovarian fibromatosis. The case is also unique being the first to describe management of these combined pathologies with cytotoxic chemotherapy.

These conditions remain rare, poorly understood and potentially life threatening. Therapeutic options are unclear and there are anecdotal reports of spontaneous regressions.[35] Although without chemotherapy it is unlikely that complete disease resolution would have occurred, response to treatment was almost certainly multi-factorial, combining the effects of cytotoxic drugs, use of steroids and good supportive care. Use of cytotoxic chemotherapy in the face of life-threatening complications appears, therefore, to offer some therapeutic benefit to these conditions.

### References

1. Enzinger, Weiss. *Soft Tissue Tumours*. 4th edn. New York: Mosby, Inc, 2001.
2. Li M, Cordon-Cardo C, Gerald WL, Rosai J. Desmoid fibromatosis is a clonal process. *Hum Pathol* 1996; 27(9): 939–43.
3. Russell P, Farnsworth A. *Surgical Pathology of the Ovaries*. 2nd edn. 1997.

4. Sakaki M, Hirokawa M, Horiguchi H, Wakatsuki S, Sano T, Izumi Y. Ovarian fibrothecoma with massive edema. *J Med Invest* 2000; 47(3–4): 148–51.
5. Lacson AG, Alrabeeah A, Gillis DA, Salisbury S, Grantmyre EB. Secondary massive ovarian edema with Meig's syndrome. *Am J Clin Pathol* 1989; 91(5): 597–603.
6. Scurry J, Allen D, Dobson P. Ovarian fibromatosis, ascites and omental fibrosis. *Histopathology* 1996; 28(1): 81–4.
7. Roth LM, Czernobilsky B. *Tumours and Tumour-like Conditions of the Ovary*. Edinburgh: Churchill Livingstone, 1985.
8. Nogales FF, Martin-Sances L, Mendoza-Garcia E, Salamanca A, Gonzalez-Nunez MA, Pardo Mindan FJ. Massive ovarian oedema. *Histopathology* 1996; 28(3): 229–34.
9. Young RH, Scully RE. Fibromatosis and massive edema of the ovary, possibly related entities: a report of 14 cases of fibromatosis and 11 cases of massive edema. *Int J Gynecol Pathol* 1984; 3(2): 153–78.
10. Rhoads JE, Terrell AW. Ovarian fibroma with ascites and hydrothorax (Meig's Syndrome). *J Am Med Assoc* 1937; 109: 1684–7.
11. Ferrario T, Karakousis CP. Retroperitoneal sarcomas: grade and survival. *Arch Surg* 2003; 138(3): 248–51.
12. Ballo MT, Zagars GK, Pollack A, Pisters PW, Pollack RA. Desmoid tumor: prognostic factors and outcome after surgery, radiation therapy, or combined surgery and radiation therapy. *J Clin Oncol* 1999; 17(1): 158–67.
13. Nuyttens JJ, Rust PF, Thomas CR Jr, Turrisi AT III. Surgery versus radiation therapy for patients with aggressive fibromatosis or desmoid tumors: A comparative review of 22 articles. *Cancer* 2000; 88(7): 1517–23.
14. Merchant NB, Lewis JJ, Woodruff JM, Leung DH, Brennan MF. Extremity and trunk desmoid tumors: a multifactorial analysis of outcome. *Cancer* 1999; 86(10): 2045–52.
15. Jelinek JA, Stelzer KJ, Conrad E, Bruckner J, Kliot M, Koh W, *et al.* The efficacy of radiotherapy as postoperative treatment for desmoid tumors. *Int J Radiat Oncol Biol Phys* 2001; 50(1): 121–5.
16. Lim CL, Walker MJ, Mehta RR, Das Gupta TK. Estrogen and antiestrogen binding sites in desmoid tumors. *Eur J Cancer Clin Oncol* 1986; 22(5): 583–7.
17. Fischer SM, Mills GD, Slaga TJ. Inhibition of mouse skin tumor promotion by several inhibitors of arachidonic acid metabolism. *Carcinogenesis* 1982; 3(11): 1243–5.
18. Sportiello DJ, Hoogerland DL. A recurrent pelvic desmoid tumor successfully treated with tamoxifen. *Cancer* 1991; 67(5): 1443–6.
19. Izes JK, Zinman LN, Larsen CR. Regression of large pelvic desmoid tumor by tamoxifen and sulindac. *Urology* 1996; 47(5): 756–9.
20. Antoniuk P, Tjandra JJ, Lavery IC. Diffuse intra-abdominal fibromatosis in association with bilateral ovarian fibromatosis and oedema. *Aust N Z J Surg* 1993; 63(4): 315–8.
21. Mukherjee A, Malcolm A, de la HM, Neal DE. Pelvic fibromatosis (desmoid)–treatment with steroids and tamoxifen. *Br J Urol* 1995; 75(4): 559–60.
22. Lanari A. Effect of progesterone on desmoid tumors (aggressive fibromatosis). *New Engl J Med* 1983; 309(24): 1523.
23. Wilcken N, Tattersall MH. Endocrine therapy for desmoid tumors. *Cancer* 1991; 68(6): 1384–8.
24. Benson JR, Mokbel K, Baum M. Management of desmoid tumours including a case report of toremifene. *Ann Oncol* 1994; 5(2): 173–7.
25. Nishida T, Ushijima K, Watanabe J, Kage M, Nagaoka S. Sclerosing peritonitis associated with luteinized thecoma of the ovary. *Gynecol Oncol* 1999; 73(1): 167–9.
26. Weiss AJ, Lackman RD. Low-dose chemotherapy of desmoid tumors. *Cancer* 1989; 64(6): 1192–4.
27. Azzarelli A, Gronchi A, Bertulli R, Tesoro JD, Baratti D, Pennacchioli E, *et al.* Low-dose chemotherapy with methotrexate and vinblastine for patients with advanced aggressive fibromatosis. *Cancer* 2001; 92(5): 1259–64.
28. Weiss AJ, Horowitz S, Lackman RD, Lackmen RD. Therapy of desmoid tumors and fibromatosis using vinorelbine. *Am J Clin Oncol* 1999; 22(2): 193–5.
29. Roche WR, du Boulay CE. A case of ovarian fibromatosis with disseminated intra-abdominal fibromatosis. *Histopathology* 1989; 14(1): 101–7.
30. Clement PB, Young RH, Hanna W, Scully RE. Sclerosing peritonitis associated with luteinized thecomas of the ovary. A clinicopathological analysis of six cases. *Am J Surg Pathol* 1994; 18(1): 1–13.
31. Frigerio L, Taccagni GL, Mariani A, Mangili G, Ferrari A. Idiopathic sclerosing peritonitis associated with florid mesothelial hyperplasia, ovarian fibromatosis, and endometriosis: a new disorder of abdominal mass. *Am J Obstet Gynecol* 1997; 176(3): 721–2.
32. Werness BA. Luteinized thecoma with sclerosing peritonitis. *Arch Pathol Lab Med* 1996; 120(3): 303–6.
33. Spiegel GW, Swiger FK. Luteinized thecoma with sclerosing peritonitis presenting as an acute abdomen. *Gynecol Oncol* 1996; 61(2): 275–81.
34. Iwasa Y, Minamiguchi S, Konishi I, Onodera H, Zhou J, Yamabe H. Sclerosing peritonitis associated with luteinized thecoma of the ovary. *Pathol Int* 1996; 46(7): 510–4.
35. Jenkins NH, Freedman LS, McKibbin B. Spontaneous regression of a desmoid tumour. *J Bone Joint Surg Br* 1986; 68(5): 780–1.
36. Raney B, Evans A, Granowetter L, Schnaufer L, Uri A, Littman P. Nonsurgical management of children with recurrent or unresectable fibromatosis. *Pediatrics* 1987; 79(3): 394–8.
37. Waddell WR, Kirsch WM. Testolactone, sulindac, warfarin, and vitamin K1 for unresectable desmoid tumors. *Am J Surg* 1991; 161(4): 416–21.
38. Patel SR, Evans HL, Benjamin RS. Combination chemotherapy in adult desmoid tumors. *Cancer* 1993; 72(11): 3244–7.
39. Okuno SH, Edmonson JH. Combination chemotherapy for desmoid tumors. *Cancer* 2003; 97(4): 1134–5.
